# Supplementary material for: ImmunoCluster provides a computational framework for the nonspecialist to profile high-dimensional cytometry data
Source: eLife. 2021 Apr 30;10:e62915. doi: 10.7554/eLife.62915 (PMC8112868; doi:10.7554/eLife.62915)
Supplement: Supplementary file 1. [file elife-62915-supp1.docx]

**Supplementary file 1.** Reference panel of anti-human antibodies for mass cytometry used by Hartmann *et al.* [1].

| **Isotope** | **Element** | **Marker** | **Clone** | **Staining step** |
| --- | --- | --- | --- | --- |
| 89 | Y | CD45 | H130 | Surface |
| 139 | La | CD235αβ/CD61 | HIR2/VI-PL2 | Surface |
| 141 | Pr | CD3 | UCHT1 | Surface |
| 142 | Nd | CD19 | HIB19 | Surface |
| 143 | Nd | CD117 | 104D2 | Surface |
| 144 | Nd | CD11b | IRCF44 | Surface |
| 145 | Nd | CD4 | RPA-T4 | Surface |
| 146 | Nd | CD8α | RPA-T8 | Surface |
| 147 | Sm | CD11c | BU15 | Surface |
| 148 | Nd | CD14 | RMO52 | Surface |
| 150 | Nd | FcεRI | AER-37 (CRA-1) | Surface |
| 151 | Eu | CD123 | 6H6 | Surface |
| 152 | Sm | γδTCR | 11F2 | Surface |
| 153 | Eu | CD45RA | HI100 | Surface |
| 154 | Sm | TIM3 | F38-2E2 | Surface |
| 156 | Gd | PD-L1 (CD274) | 29E.2A3 | Surface |
| 158 | Gd | CD27 | L128 | Surface |
| 160 | Gd | Tbet | 4B10 | Intracellular |
| 161 | Dy | CD152 (CTLA-4) | 14D3 | Intracellular |
| 162 | Dy | FoxP3 | PCH101 | Intracellular |
| 163 | Dy | CD33 | WM53 | Surface |
| 164 | Dy | CD45RO | UCHL1 | Surface |
| 165 | Ho | CD127 | A019D5 | Surface |
| 167 | Er | CCR7 (CD197) | G043H7 | Surface |
| 168 | Er | Ki-67 | B56 | Intracellular |
| 169 | Tm | CD25 | 2A3 | Surface |
| 170 | Er | TCR Va24-Ja18 | 6B11 | Intracellular |
| 172 | Yb | CD38 | HIT2 | Surface |
| 174 | Yb | HLA-DR | L243 | Surface |
| 175 | Lu | PD-1 | EH12.2H7 | Surface |
| 176 | Yb | CD56 | NCAM16.2 | Surface |
| 209 | Bi | CD16 | 3G8 | Surface |
