# Supplementary material for: ImmunoCluster provides a computational framework for the nonspecialist to profile high-dimensional cytometry data
Source: eLife. 2021 Apr 30;10:e62915. doi: 10.7554/eLife.62915 (PMC8112868; doi:10.7554/eLife.62915)
Supplement: Supplementary file 2. [file elife-62915-supp2.docx]

**Supplementary file 2.** Reference panel of anti-human antibodies for the head and neck cancer (HNSCC) imaging mass cytometry experiment.

| **Isotope** | **Element** | **Channel** | **Marker** |
| --- | --- | --- | --- |
| 150 | Nd | C1 | PDL-1 |
| 152 | Sm | C2 | CD45 |
| 156 | Gd | C3 | CD4 |
| 158 | Gd | C4 | E-Cadherin |
| 159 | Tb | C5 | CD68 |
| 161 | Dy | C6 | CD20 |
| 162 | Dy | C7 | CD8α |
| 165 | Ho | C8 | PD-1 |
| 168 | Er | C9 | Ki-67 |
| 170 | Er | C10 | CD3 |
| 191 | Ir | C11 | DNA |
| 193 | Ir | C12 | DNA |
