# Supplementary material for: ImmunoCluster provides a computational framework for the nonspecialist to profile high-dimensional cytometry data
Source: eLife. 2021 Apr 30;10:e62915. doi: 10.7554/eLife.62915 (PMC8112868; doi:10.7554/eLife.62915)
Supplement: Supplementary file 3. [file elife-62915-supp3.docx]

**Supplementary file 3.** Reference panel of anti-human antibodies for the diffuse large B-cell lymphoma (DLBCL) imaging mass cytometry experiment.

| **Isotope** | **Element** | **Marker** | **Dilution*** |
| --- | --- | --- | --- |
| 141 | Pr | αSMA | 1:2000 |
| 144 | Nd | CD74 | 1:100 |
| 146 | Nd | CD16 | 1:200 |
| 147 | Sm | CD68 | 1:500 |
| 150 | Nd | PD-L1 | 1:100 |
| 151 | Eu | CD31 | 1:200 |
| 152 | Sm | CD45 | 1:500 |
| 155 | Gd | FOXP3 | 1:1000 |
| 156 | Gd | CD4 | 1:200 |
| 161 | Dy | CD20 | 1:400 |
| 162 | Dy | CD8 | 1:400 |
| 165 | Ho | PD1 | 1:100 |
| 166 | Er | CD45RA | 1:2000 |
| 167 | Er | Granzyme B | 1:100 |
| 168 | Er | Ki-67 | 1:1000 |
| 170 | Er | CD3 | 1:400 |
| 173 | Yb | CD45 RO | 1:1000 |
| 175 | Lu | CD11c | 1:400 |
| 176 | Yb | β2 microglobulin | 1:200 |
| 191 | Ir | nuclei | 1:4000 |

*Dilution factors for antibody cocktail described in Appendix 2.
