# Supplementary material for: ImmunoCluster provides a computational framework for the nonspecialist to profile high-dimensional cytometry data
Source: eLife. 2021 Apr 30;10:e62915. doi: 10.7554/eLife.62915 (PMC8112868; doi:10.7554/eLife.62915)
Supplement: Supplementary file 4. [file elife-62915-supp4.docx]

**Table S4.** D*iffcyt* computational framework [2] output for differential discovery analysis for identified cell clusters in the Hartmann *et al.* [1] in LMC data.

| **cluster_id** | **logFC** | **logCPM** | **LR** | **p_val** | **p_adj** |
| --- | --- | --- | --- | --- | --- |
| B Cell Naive | 4.006128 | 14.95084 | 10.13927 | 0.001451 | 0.018159 |
| B Cell Memory | 3.384943 | 10.83426 | 10.0624 | 0.001513 | 0.018159 |
| CD4^+^ T Naive | 2.717506 | 13.46515 | 8.242607 | 0.004092 | 0.032735 |
| NKT Cell | 2.294813 | 13.02842 | 5.133276 | 0.023471 | 0.140828 |
| B Cell Plasmablast | 2.794889 | 13.58889 | 4.201374 | 0.040391 | 0.193878 |
| cDC | 1.456054 | 14.82018 | 3.441664 | 0.063572 | 0.254287 |
| pDC | 1.349146 | 11.86775 | 2.853596 | 0.09117 | 0.312582 |
| CD4^+^ T Effector memory | 0.8481 | 15.3479 | 2.097111 | 0.147578 | 0.437681 |
| gd T Cell | 1.072 | 14.31152 | 1.848994 | 0.1739 | 0.437681 |
| CD8^+^ Central memory | -1.00903 | 14.68047 | 1.758226 | 0.184846 | 0.437681 |
| CD8^+^ T EMRA | 1.077264 | 15.54702 | 1.637974 | 0.200604 | 0.437681 |
| CD8^+^ T Naive | 0.825672 | 13.87339 | 1.375561 | 0.240859 | 0.481717 |
| Monocyte CD14^+^CD16^-^ | -0.56723 | 18.51568 | 1.213405 | 0.270659 | 0.499678 |
| Monocyte CD14^-^CD16^+^ | 0.612952 | 13.14178 | 0.522886 | 0.469613 | 0.805051 |
| CD4^+^ Treg | -0.34213 | 13.28199 | 0.318272 | 0.572648 | 0.842639 |
| HLA_DR^-^CD16^+^ | -0.46549 | 12.50334 | 0.286002 | 0.592794 | 0.842639 |
| Basophil | 0.448909 | 11.98577 | 0.279743 | 0.59687 | 0.842639 |
| NK Cell CD16^+^ | 0.297575 | 15.60477 | 0.178402 | 0.67275 | 0.860578 |
| CD8^+^ T Effector memory | 0.276937 | 16.77325 | 0.168676 | 0.681291 | 0.860578 |
| NK Cell CD16- | -0.1954 | 16.14487 | 0.115805 | 0.73363 | 0.880356 |
| Mast Cell | 0.179125 | 13.66913 | 0.065481 | 0.798033 | 0.912038 |
| T Cell Unassigned | 0.138984 | 14.34233 | 0.02963 | 0.863331 | 0.941816 |
| CD4^+^ T Central memory | -0.02508 | 15.72401 | 0.002204 | 0.962556 | 0.982714 |
| Monocyte CD14^+^CD16^+^ | -0.01626 | 14.63405 | 0.000469 | 0.982714 | 0.982714 |

* Comparison between GvHD and none.
